# An Assessment of Dispersion-Corrected DFT Methods for Modeling Nonbonded Interactions in Protein Kinase Inhibitor Complexes

**DOI:** 10.3390/molecules29020304

**Published:** 2024-01-06

**Authors:** Yan Zhu, Saad Alqahtani, Xiche Hu

**Affiliations:** 1 Department of Chemistry and Biochemistry, University of Toledo, Toledo, OH 43606, USA; yan.zhu2@rockets.utoledo.edu (Y.Z.); salqahtani2@ksu.edu.sa (S.A.); 2 Department of Chemistry, King Saud University, Riyadh 12372, Saudi Arabia

**Keywords:** dispersion-corrected DFTs, benchmarking, protein kinase inhibitors, motifs of nonbonded interactions, CCSD(T)/CBS reference, molecular recognition

## Abstract

Accurate modeling of nonbonded interactions between protein kinases and their small molecule inhibitors is essential for structure-based drug design. Quantum chemical methods such as density functional theory (DFT) hold significant promise for quantifying the strengths of these key protein–ligand interactions. However, the accuracy of DFT methods can vary substantially depending on the choice of exchange–correlation functionals and associated basis sets. In this study, a comprehensive benchmarking of nine widely used DFT methods was carried out to identify an optimal approach for quantitative modeling of nonbonded interactions, balancing both accuracy and computational efficiency. From a database of 2139 kinase-inhibitor crystal structures, a diverse library of 49 nonbonded interaction motifs was extracted, encompassing CH–π, π–π stacking, cation–π, hydrogen bonding, and salt bridge interactions. The strengths of nonbonded interaction energies for all 49 motifs were calculated at the advanced CCSD(T)/CBS level of theory, which serve as references for a systematic benchmarking of BLYP, TPSS, B97, ωB97X, B3LYP, M062X, PW6B95, B2PLYP, and PWPB95 functionals with D3BJ dispersion correction alongside def2-SVP, def2-TZVP, and def2-QZVP basis sets. The RI, RIJK, and RIJCOSX approximations were used for selected functionals. It was found that the B3LYP/def2-TZVP and RIJK RI-B2PLYP/def2-QZVP methods delivered the best combination of accuracy and computational efficiency, making them well-suited for efficient modeling of nonbonded interactions responsible for molecular recognition of protein kinase inhibitors in their targets.

## 1. Introduction

Protein kinases are a large family of enzymes that play a central role in cellular regulation by catalyzing the transfer of the γ-phosphate group from ATP to their target substrates. This process, known as protein phosphorylation, is a fundamental regulatory mechanism for numerous cellular functions, including metabolism, cell growth and division, differentiation, apoptosis, and gene expression [1]. Dysregulation of protein kinase activity has been implicated in many human diseases, such as cancer, inflammation, diabetes, nervous system disorders, and cardiovascular disease [2]. Cancer cells, in particular, rely on protein phosphorylation by specific kinases to promote abnormal proliferation, metastasis, angiogenesis, and survival. Over 400 diseases are thought to be linked to protein kinases, either directly or indirectly [3]. This has spurred considerable interest in developing small molecule protein kinase inhibitors (PKIs) capable of modulating kinase function for therapeutic benefit [4]. The majority of PKIs are ATP binding site competitive inhibitors, which are the primary targets of this research.

Ever since the first approval of the PKI drug imatinib by the FDA for the treatment of chronic myeloid leukemia in 2001, ATP binding site competitive PKIs have emerged as one of the most significant therapeutic agents in the 21st century for treating a variety of diseases, including cancer, heart disease, and diabetes [4]. However, many challenges remain in PKI drug discovery, such as drug resistance and off-target-mediated toxicity [2,5,6]. The key to overcoming these challenges lies in the targeted molecular design of more potent and selective PKIs. To do so, an atomic level understanding of the molecular recognition between PKIs and their specific kinase targets is required [6].

It is nonbonded interactions that mediate molecular recognition between PKIs and their target protein kinases, as in all ligand–protein complexes [7,8]. Traditionally, the consideration of nonbonded interactions mainly included hydrogen bonding and salt bridge interactions. However, in recent years, more and more evidence suggests that π-moiety involved interactions, such as π–π stacking interactions [9], CH–π interactions [10], cation–π interactions [11], are just as important as hydrogen bonding and salt bridges [6,12,13]. As is established in Ref. [12], hereinafter, all these π-moiety involved interactions will be collectively termed “nonbonded π-interactions”.

To better understand molecular recognition of PKIs in the binding pockets of protein kinases, we are establishing a library of 3D binding motifs between PKIs and their interacting residues inside protein kinases. In a previous study [6], we carried out a large-scale data mining of the Protein Data Bank (PDB), which resulted in the establishment of a database of 2139 non-redundant high-resolution X-ray crystal structures of PKIs bound to protein kinases. As you will see in the Results and Discussion section, care is taken to ensure that the library of 3D binding motifs samples are representative of all major modes of nonbonded interactions occurring in the database of 2139 PKI bound protein kinases, including hydrogen bonding, salt bridge, CH–π interaction, π–π stacking interactions, and cation–π interaction.

Subsequently, intermolecular interaction energies for all motifs in the library were calculated at the highest level of electronic structure theory currently available, i.e., the coupled cluster methods with single, double, and perturbative triple excitations CCSD(T) at complete basis set (CBS) [14]. Besides gaining mechanistic insights on molecular recognition of PKIs in protein kinases, this work has two practical implications: (1) The library of 3D motifs based on real life PKI-bound complexes will provide medicinal chemists with working structure motifs that can guide future design of next generation PKIs; and (2) The magnitudes of intermolecular interaction energies for all motifs of nonbonded intermolecular interactions calculated at the advanced CCSD(T)/CBS level of theory will serve as references for theoreticians to benchmark existing or newly developed methods of intermolecular interaction energy calculations. The latter is exactly what we carried out in the second half of this article: benchmarking of the widely applied density functional theory (DFT) methods against CCSD(T)/CBS.

Nonbonded interactions are essentially a juxtaposition of several elements, including electrostatic interactions, exchange repulsion interactions, induction, and dispersion forces. Of these, dispersion forces constitute the dominant attractive forces between neutral molecules [15,16]. Dispersion forces arise from the mutual correlation of electrons that belong to interacting monomers (intermolecular correlation effects). For a proper treatment of dispersions, post-Hartree–Fock methods that include electron correlation corrections are needed. Therefore, for the study of biological macromolecular systems, applicable wavefunction-based post-Hartree–Fock methods are quite limited due to their large system size. Over the years, second-order Møller–Plesset perturbation (MP2) has been the working horse for calculations of nonbonded interactions because of its efficiency and convenience [17]. The highly accurate CCSD(T) method has been widely applied to small molecular systems. However, its applicability to large biological systems is severely limited due to the extremely high demand of the CCSD(T) method for both CPU time and core memory until recently.

The DFT method is an alternative approach that provides an unrivaled practical balance of accuracy and computational cost. Although the DFT method has been widely applied to study covalently bonded molecules, its application to nonbonded intermolecular interactions is limited due to its inability to capture long-range correlation effects [18]. As a result, typical pure DFT functionals do not take dispersion interactions into consideration. Fortunately, a new generation of DFT methods have been developed with dispersion correction, making it possible to apply DFT methods for analyzing biological systems that are interacting mainly through dispersion interactions [16]. However, the accuracies of dispersion corrected DFTs varied widely. In this work, we performed a systematic benchmarking of nine widely used exchange–correlation functionals, BLYP [19,20], TPSS [21], B97 [22], ωB97X [23], B3LYP [24,25], M062X [26], PW6B95 [27], B2PLYP [28] and PWPB95 [29]. Those functionals span the entire spectrum of the Jacob’s ladder, including two (meta-)GGAs, five (meta-)hybrid functionals, and two double-hybrid functionals. With the inclusion of the D3 level dispersion correction developed by Grimme [30], their performance is calibrated against the ‘gold standard’ CCSD(T) method for a diverse set of 49 motifs of nonbonded interactions extracted from X-ray structures of kinase–inhibitor complexes. Here, we aim at determining the best performing dispersion-corrected DFT method in terms of not only the accuracy but also the computational efficiency. Our objective is to arrive at a recommendation on the best performing DFT protocol that is applicable to routine modeling of protein–ligand binding in kinases.

Another important consideration for the theoretical treatment of nonbonded interactions is the judicious selection of a basis set that effectively balances accuracy and efficiency. Two widely used series of basis sets in electronic structure methods, which take into account electron correlations, are the def2 series developed by Ahlrichs and Weigend [31] and Dunning’s correlation-consistent family of basis sets (cc-pVXZ) [32]. The CC-pVXZ basis sets, known for their correlation consistent design, were selected for the CCSD(T) calculations. The def2 series of basis sets were employed in the DFT methods for their balanced accuracy and computational efficiency. The latter is achieved through density fitting, also known as resolution of the identity (RI) or fitting auxiliary basis sets. Density fitting has been seamlessly incorporated into calculations that employ the def2 series of basis sets in the ORCA program [33]. In this work, def2-SVP, def2-TZVP, and def2-QZVP basis sets were tested in all DFT methods for a systematical study of basis set dependence.

Historically, benchmarking of theorical approaches for nonbonded interaction energy calculations has been based on prototypical model systems of nonbonded interactions [34,35]. The latter are typically at equilibrium geometries or artificially scaled distances between the nonbonded interacting pairs [36]. This is problematic because, for a given theoretical method, the accuracy of the calculated interaction energy is known to be sensitively geometry (distance) dependent. In contrast, the 3D motifs of nonbonded interactions in our study were obtained directly from actual real life PKI-bound protein complexes. Thus, it is our expectation that the winner of this benchmarking study (i.e., the top performing DFT approach) will be better suited for molecular modeling of PKI binding with protein kinases in any future structure-based drug design of potent and selective PKIs.

As noted earlier, the CCSD(T) interaction energies not only serve as a benchmark for this study but also offer a valuable resource for future development and validation of methods for calculating intermolecular interactions. Moreover, the established library of 3D nonbonded interaction motifs represents a significant contribution in its own right, providing a valuable dataset for future studies exploring protein–ligand recognition and beyond.

## 2. Results and Discussion

### 2.1. Library of 3D Motifs of Nonbonded Interactions

In order to construct a comprehensive library of three-dimensional (3D) binding motifs to model the interactions between PKIs and protein kinases, we meticulously selected representative motifs of pairwise nonbonded interactions between PKIs and their interacting residues inside the ATP binding pocket of protein kinases. In a previous study, we carried out a large-scale data mining of the PDB, which resulted in the establishment of a database of 2139 non-redundant high-resolution X-ray crystal structures of kinase–inhibitor complexes [6]. Based on this database, 49 unique motifs of nonbonded interaction pairs between PKIs and their interacting residues were extracted. These represent key modes of nonbonded interactions, including 13 CH–π interactions, 12 π–π stacking interactions, 8 cation–π interactions, 8 hydrogen bonding, and 8 salt bridge interactions. The selection of these motifs was governed by two main criteria. The first criterion prioritizes protein kinases that are the targets of disease treatment. For example, multiple cyclin-dependent kinases (CDKs) were included, owing to their crucial role in cell cycle regulation and their status as high-priority targets for kinase inhibitor development. The second criterion ensures that these 49 motifs comprehensively cover the diverse spectrum of possible intermolecular interactions, such as hydrogen bonding, salt bridge, and all non-bonded π-interactions, in proportion to their occurrence frequencies in the database of 2139 PKI-Protein complexes [6].

Table 1 provides a full list of relevant details on all 49 motifs. For easy reference, a motif ID is assigned in column 1. Column 2 defines the mode of nonbonded interactions. Column 3 lists the PDB IDs for the PKI-bound complexes from which the binding motifs are extracted. Column 4 lists the interacting pairs, with PKIs labeled by the three letter ligand IDs of the corresponding PDB file and their interacting residues labeled by the residue IDs. Geometrical features for the interacting pairs are given in Columns 5 and 6. For hydrogen bonding pairs, two sets of H-bond angles and distances are listed since there are dual hydrogen bonds in all motifs except motif 40. For all other modes of nonbonded interactions, the closest atom to atom distance between the PKI and its interacting residue is tabulated. For π–π stacking interactions, the angle is measured between the two π planes of the interacting partners. The last column lists the reference in which the X-ray crystal structure was reported.

**Figure 1:**
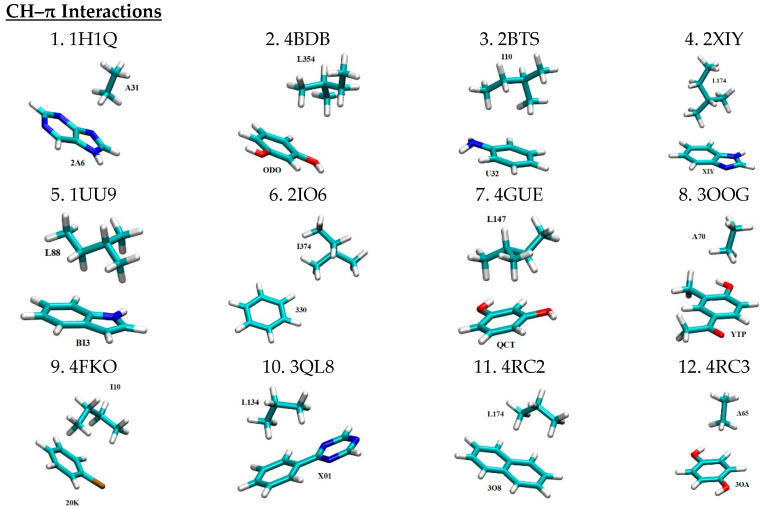

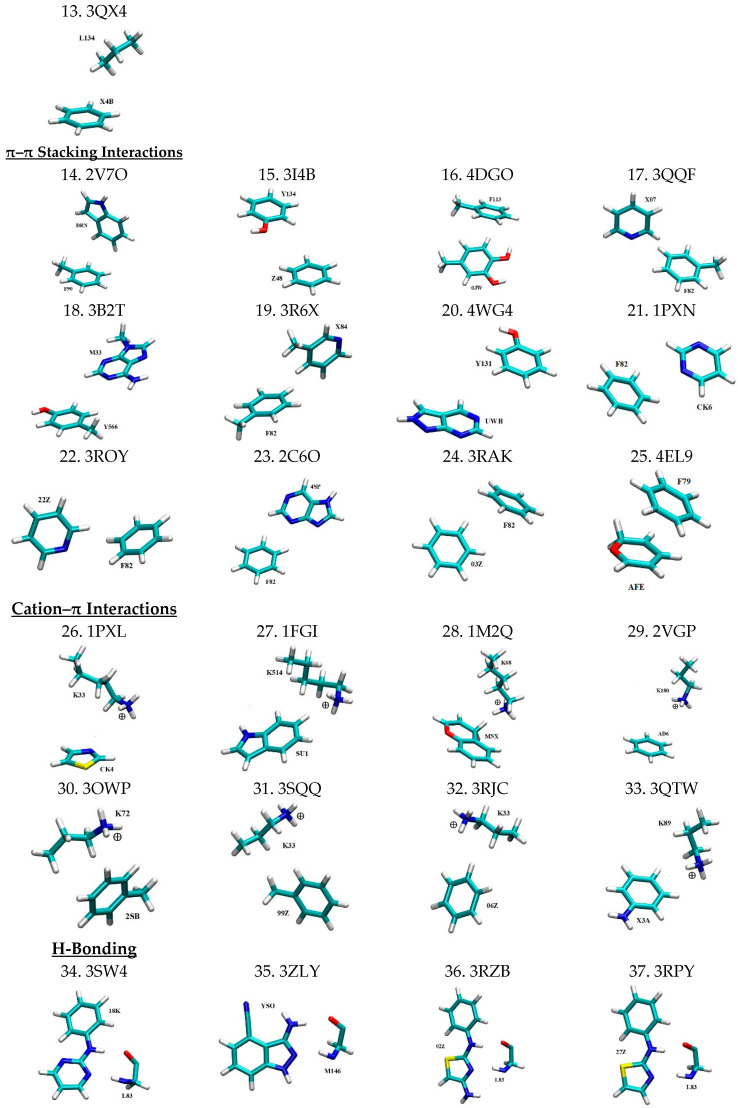

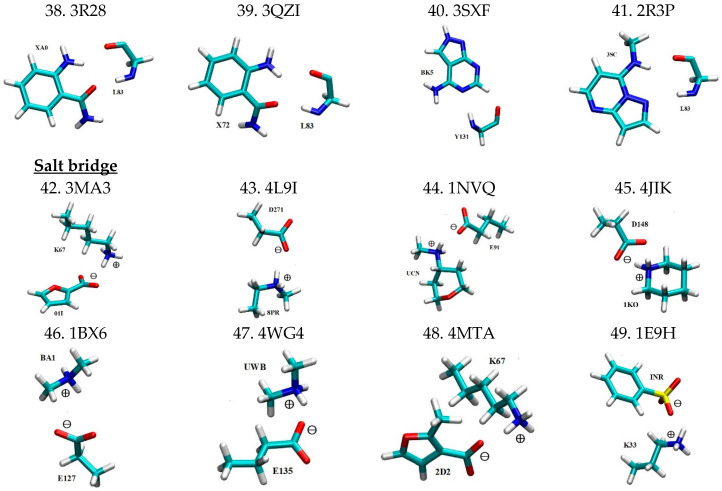
Motifs of nonbonded interactions between PKIs (labeled by the three-letter ligand IDs) and their interacting residues (labeled by the residue IDs) in a licorice representation with carbon, oxygen, nitrogen, sulfur, hydrogen and chlorine atoms colored in cyan, red, blue, yellow, white and brown, respectively. The electric charges of constituting monomers are labeled for motifs of cation-π and salt bridge interactions. All motifs have a spin multiplicity of 1. This figure is produced with the program VMD 1.9.3 [65].

Three dimensional structures for all 49 motifs of nonbonded interaction pairs between PKIs and their interacting residues are depicted in Figure 1 in the same motif count order as in Table 1. The four letter codes after the motif count represent the PDB IDs for the PKI bound complexes from which the binding motifs are extracted. For each binding motif, the PKI and its interacting residue are labeled by the three-letter ligand ID and the residue ID number of the corresponding PDB file, respectively. Given the large size of PKI molecules, only the functional group of a given PKI that directly interacts with the protein was kept as part of the 3D motif. The coordinates of all the non-hydrogen atoms were extracted from their respective PDB files. Hydrogen atoms were patched to satisfy the valency by means of a geometry optimization calculation at the HF/6-31+G levels using the Gaussian 09 program [64]. It should be cautioned that the X-ray crystal structures from which the motifs were extracted may potentially have been distorted by crystal packing. However, the extent of such distortion is a matter of hot debate, with some seeing it as minor adjustments while others view it as significant.

In a previous study, we established a pharmacophore model for ATP-competitive kinase inhibitors [6]. According to this pharmacophore model, PKIs are a special class of small molecules that feature a scaffold of one or more aromatic rings that are linked with one or more hydrophilic functional groups. The former has the structural role of acting as a scaffold and the functional role of participating in nonbonded π-interactions (i.e., π–π stacking interactions, CH–π interactions, cation–π interactions, and XH–π interactions (XH = NH, SH, OH)) with hydrophobic regions I, II, and III (see Ref. [6] for details), and the adenine region. The latter ensure water solubility and form hydrogen bonds with the hinge region and other hydrophilic residues of the ATP binding pocket. This pharmacophore model, to a large extent, functions as a blueprint to guide the selection of the 49 binding motifs sampled here. As shown in Figure 1, in all 13 motifs of CH–π interactions, aromatic rings of PKIs interact with the CH groups of the aliphatic residues Ala, Leu, and Ile. In the case of π–π stacking interactions, aromatic rings of PKIs interact with aromatic residues Phe, Tyr, and Trp. As can be seen from Table 1, a wide spectrum of π–π stacking angles are sampled, ranging from parallel displaced to T-shaped configuration, and everything in between. In this study, the coordinates for all motifs of nonbonded interactions were directly extracted from the experimentally determined X-ray crystal structures. Inside the protein, the conformation of the interacting pairs is constrained by a multitude of forces. As a results, the extracted motifs of non-bonded interactions are generally not in energetically optimal conformations that are achievable in the gas phase, such as the perfect T-shaped or parallel-displaced configurations for π–π stacking interaction pairs [13]. Motifs of cation–π interaction feature aromatic rings of PKIs interacting with the positively charged side chains of the basic residue Lys. In motifs of hydrogen bonding pairs, the backbone N and O atoms of residues in the hinge region are involved in dual hydrogen bonding with H bond donors and acceptors of PKIs. This mirrors a major hydrogen bonding pattern for molecular recognition of FDA approved small molecule protein kinase inhibitor drugs in protein kinases [12]. For motifs of salt bridges, positively charged basic residue Lys and negatively charged acidic residues (Asp and Glu) in protein kinases interact with oppositely charged functional groups of PKIs, respectively.

### 2.2. Energies of Intermolecular Interactions Calculated at the CCSD(T)/CBS Level

The strengths of the intermolecular interaction energies for all 49 motifs were calculated at both the gas phase and the solution phase. The former serves as the reference for benchmarking the DFT methods in the next subsection. The latter aims at a realistic evaluation of the strengths of the intermolecular interactions in the aqueous media where the actual biological interactions occur. Table 2 lists both the gas phase ( ${∆E}_{CCSD(T)}^{g}$ ) and the solution phase ( ${∆E}_{int}^{aq}\;$ ) intermolecular interaction energies for all 49 motifs. The gas phase interaction energies were calculated at the CCSD(T) level and extrapolated to the complete basis limit (CBS) via the two-point basis set extrapolation scheme [66,67], with the basis set superposition error (BSSE) corrections (see Section 3 for details). Following Equation (3), the CCSD(T)/CBS level interaction energy was obtained by $∆E_{\mathrm{CBS}}^{\mathrm{CCSD}(T)}=∆E_{\mathrm{CBS}}^{\mathrm{MP}2}+(∆E^{\mathrm{CCSD}(T)}-∆E^{\mathrm{MP}2}){|}_{\mathrm{aug}-\mathrm{cc}-\mathrm{pVDZ}}$ . The first term $∆E_{\mathrm{CBS}}^{\mathrm{MP}2}\;$ defines the MP2 level interaction energy with complete basis set, and was evaluated using Equation (4) with the cc-pVTZ and cc-pVQZ basis sets. The second term, commonly referred to as “coupled cluster correction” in the literature [68], was calculated using the aug-cc-pVDZ basis set. Given the demanding scaling of the CCSD(T) method with the system size, the CCSD(T)/aug-cc-pVDZ protocol employed here represents what is computationally feasible currently for the large size motifs studied. It is important to note that achieving convergence for the “coupled cluster correction” term might necessitate a basis set more extensive than aug-cc-pVDZ [68]. In the case of benzene dimer, for example, the “coupled cluster correction” term for π–π stacking interactions evaluated at the CCSD(T)/aug-cc-pVDZ level deviates from that of “best estimate” CCSD(T)/CBS by approximately 0.1 kcal/mol [68]. The solution phase interaction energies were obtained indirectly by means of a thermodynamic cycle according to Equation (5) (see Theory and Methods): ${∆E}_{int}^{aq}={∆E}_{CCSD(T)}^{g}+{∆E}_{Deh}$ . The dehydration energy ${∆E}_{Deh}$ itself was calculated utilizing the SM5.42R solvation model of Cramer and Truhlar [69].

As shown in the fifth column of Table 2, the pairwise interactions between PKIs and their targets are energetically favorable in the gas phase; all the calculated interaction energies are negative (attractive). For example, hydrogen bonding interactions exhibit energies ranging from −7.0 to −3.8 kcal/mol, with an average of −5.5 kcal/mol. The energies for salt bridge interactions span from an extraordinary −115.9 to −68.2 kcal/mol, averaging at −99.5 kcal/mol. Non-bonded π-interactions have a smaller magnitude, with an average gas-phase interaction energies of −2.1 kcal/mol, −1.8 kcal/mol, and −4.5 kcal/mol for CH–π interaction, π–π stacking interactions, and cation–π interactions, respectively.

The last column of Table 2 presents the solution phase interaction energies for all 49 motifs, which sheds some light on the molecular recognition of PKIs in protein kinases. All modes of nonbonded interactions in the 49 motifs display a favorable (negative) interaction energy, making an energetically stabalizing contribution to the binding of PKIs with protein kinases. Although the dispersion forces dominated CH–π interactions and π–π stacking interactions are relatively weak in the gas phase in comparison with polar and charged interactions (hydrogen bonding, salt bridge, and cation–π interactions), there is a much lower (or near zero) energetic cost of dehydration involved. As a result, significant interaction strengths remain in the aqueous phase for both CH–π interactions and π–π stacking interactions. This agrees with our published findings that, in addition to hydrogen bonding, aromatic rings of PKIs function as important molecular determinants for the binding of PKIs with protein kinases. From the perspective of aromatic rings, hydrophobic regions I, II, and III (see Ref. [6] for details), plus the adenine region, are the most relevant. Those four regions are loaded with hydrophobic residues that can participate in nonbonded π-interactions (i.e., π–π stacking interactions, CH–π interactions). The aliphatic residues Ala, Val, Leu, and Ile in the hydrophobic regions of the ATP binding site can form CH–π interactions with aromatic rings of PKIs. Additionally, aromatic residues Phe, Tyr, and Trp can form π–π stacking interactions with aromatic rings of PKIs.

It is worth noting, from the perspective of methodology, the importance of including dehydration energy in any theoretical treatment of nonbonded interactions. For example, one particularly noteworthy observation involves the strength of salt bridge interactions, which feature very strong gas-phase interaction energies of around −100 kcal/mol. While these energies might appear extremely strong, it becomes evident that the dehydration energy ${∆E}_{Deh}$ is also substantial, roughly around 100 kcal/mol. Consequently, the average intermolecular interaction energies reduce from −99.5 kcal/mol in the gas phase to −4.4 kcal/mol in the solution phase for salt bridge interactions. The same can be said about other motifs of nonbonded interactions involving polar or charged species. The average dehydration energies are 2.9 and 3.6 kcal/mol for cation–π interactions and hydrogen bonding, respectively. This underlines the necessity of incorporating desolvation energy corrections in any theoretical calculations aiming for a realistic modeling of ligand binding in proteins.

### 2.3. Benchmarking of DFT Methods

Based on the gas phase interaction energies of 49 motifs of nonbonded interactions calculated at the CCSD(T)/CBS level above, the performance of BLYP, TPSS, B97, ωB97X, B3LYP, M062X, PW6B95, B2PLYP and PWPB95 functionals was benchmarked. As shown in Table 3, these nine exchange-correction functionals span the entire spectrum of the Jacob’s ladder, featuring two (meta-)GGAs, five (meta-)hybrid functionals, and two double-hybrid functionals. Given the dual objectives of our performance benchmarking, i.e., accuracy and efficiency (see the Introduction section), different algorithms of implementation for several functionals are also evaluated. For functionals BLYP, TPSS, B97, B2PLYP, and PWPB95, the Resolution of Identity (RI) approximation (also called Density Fitting) [70] is implemented. In addition, the PW6B95 functional is implemented with the RIJCOSX approximation that uses density fitting for the Coulomb (J) integrals and numerical chain-of-sphere integration for the HF Exchange integrals (COSX) [71]. For efficiency, B2PLYP and PWPB95 functionals were also implemented with the resolution of identity (RI) approximation for the perturbation step and RIJK (RI approximation is applied to both Coulomb and HF Exchange integrals) [72] for the HF-SCF step. Basis set dependence was also studied systematically. For this purpose, the Ahlrichs def2 basis set family [31,73] was employed, including def2-SVP (split valence), def2-TZVP (triple zeta valence), and def2-QZVP (quadruple zeta valence).

Gas phase interaction energies for all 49 motifs of nonbonded interactions were calculated according to Equation (1) for all nine exchange–correlation functionals listed in Table 3. In order to compensate for the lack of adequate account of dispersion interactions, the D3 level dispersion correction [30] is added for all DFT calculations. BSSE was corrected by the Boys and Bernardi Counter Poise Method [79]. The resulting gas phase interaction energies for each of the nine dispersion-corrected DFT methods, in combination with basis sets def2-SVP, def2-TZVP, and def2-QZVP are tabulated in Tables S1–S3 of the Supplementary Material section, respectively. It is worth noting that, for functionals with various numerical algorithms of implementation, i.e., RI, RIJCOSX, and RI RIJK, there was no noticeable difference in the calculated interaction energies from those calculated with their corresponding original functionals. Hence, interaction energies are presented only using the original functionals in Tables S1–S3. The same applies to all the subsequent data analyses presented in all the tables and figures hereafter. As can be seen from the Supplementary Tables S1–S3, a majority of the DFT methods, with dispersion corrections, yielded interaction energies that are either identical or closely aligned with those obtained from CCSD(T)/CBS calculations in the gas phase. This close agreement emphasizes the potential utility of specific dispersion-corrected DFT methods as accurate yet computationally efficient tools for modeling these critical biological interactions.

To conduct an in-depth assessment of the performance of the various DFT methods and basis sets under consideration, we employed a series of statistical metrics for a comparative analysis against the benchmark CCSD(T)/CBS calculations. Specifically, we calculated the Root Mean Square Deviation (RMSD), Mean Unsigned Error (MUE), Average Signed Error (AVG), and the Maximum Deviation (MAX). RMSD serves as a widely accepted metric in computational chemistry for performance evaluation, facilitating a meaningful comparison between DFT-derived interaction energies and those ascertained via CCSD(T) calculations. The MUE metrics offer additional insights into the primary discrepancies associated with the DFT methods relative to the benchmark CCSD(T) values.

Table 4 elucidates the errors associated with the nine DFT methods in relation to the benchmark CCSD(T)/CBS calculations. Overall, the MAE values are slightly less than the RMSD values. This is not unexpected since RMSD penalizes large discrepancies more severely while offering an excellent measure of overall accuracy. Conversely, MAE, though less prone to outliers, overlooks the direction of errors (over/underestimation) and does not penalize large errors as heavily as RMSD. The AVG values are even smaller. Although AVG provides a simple measure of the error’s central tendency, it is easily skewed by outliers and lacks information about the error distribution. The MAX% values are quite large, which should not be a cause of alarm. That is because %MAX highlights the worst-case scenario for individual predictions. To a large extent, the small magnitude of interaction energies for several modes of nonbonded interactions (see Table 2 and Supplementary Tables S1–S3) are responsible for the large discrepancy in MAX%. So, we will focus on the RMSD values for our error analysis. As can be seen from Table 4, the def2-QZVP basis set consistently outperformed its def2-TZVP and def2-SVP counterparts. Notably, the most accurate RMSD value recorded was 0.46 kcal/mol, achieved using the B3LYP functional in combination with the def2-QZVP basis set. The B3LYP/def2-TZVP combination yielded second best RMSD of 0.50 kcal/mol. The third best RMSD value of 0.51 kcal/mol belongs to the double hybrid functional B2PLYP with the def2-QZVP basis set. Interestingly, for a subset of DFT methods—namely ωB97X, TPSS, B97, and PW6B95—the def2-QZVP and def2-TZVP basis sets yielded nearly identical RMSD values. However, it is crucial to point out that these RMSD values were considerably higher than those produced by the B3LYP and B2PLYP methods.

**Figure 2:**
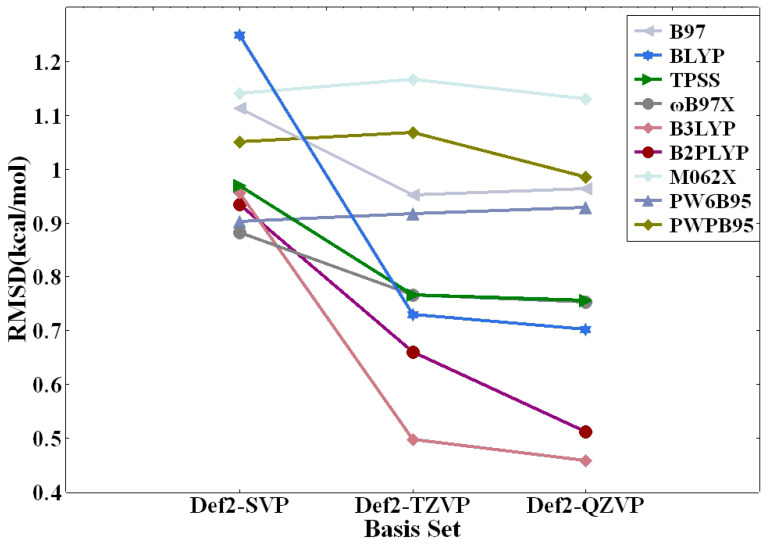
Basis set dependence of RMSD of calculated intermolecular interaction energies (in kcal/mol) for the nine DFT methods against those of CCSD(T)/CBS.

The basis set dependence is further illustrated in Figure 2. As depicted, the performance of most DFT methods is closely tied to the sizes of the basis sets employed. Generally speaking, the larger def2-QZVP basis set delivers superior results when compared to the def2-TZVP basis set, which in turn outperforms the more diminutive def2-SVP basis set across a majority of the DFT methods tested. While there are a few anomalies to this trend, it is worth noting that the disparities in RMSD values calculated at the def2-QZVP and def2-TZVP levels are negligible. This analysis accentuates the importance of carefully selecting the appropriate basis set for electronic structure calculations, especially those focused on delineating complex biological interactions. The demonstrated improvement in performance with increasing basis set size suggests that for studies requiring a high degree of accuracy, the use of larger basis sets such as def2-QZVP is recommended, although the computational cost must also be considered in the selection process.

**Figure 3:**
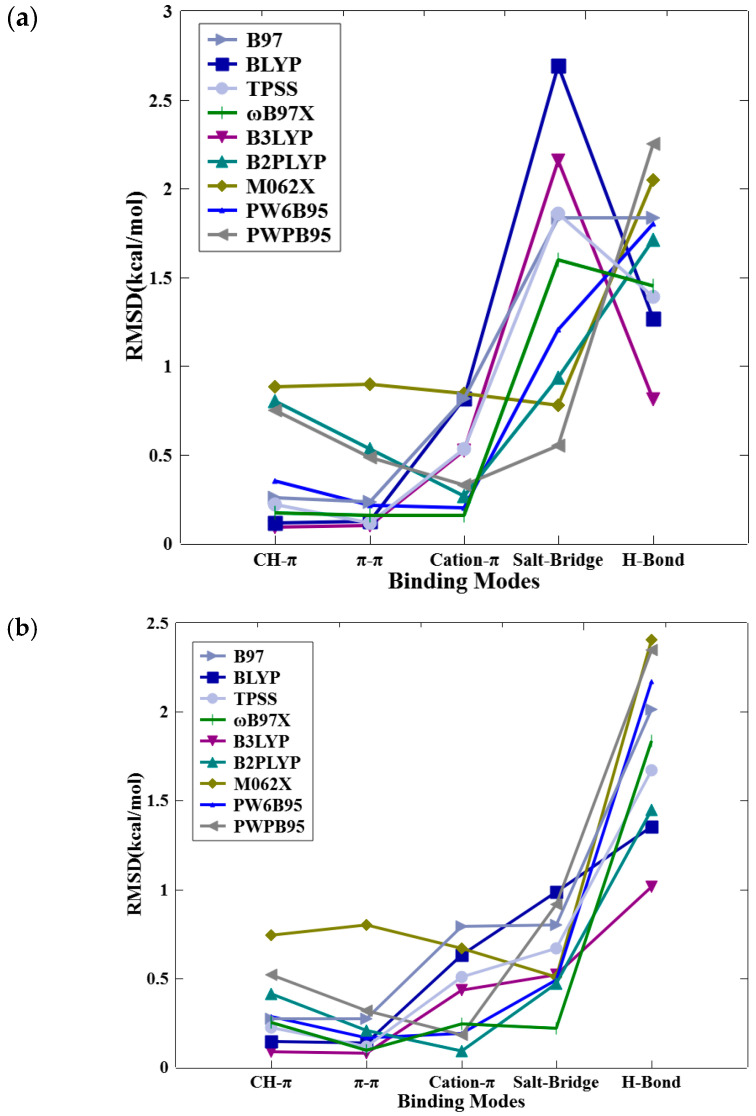

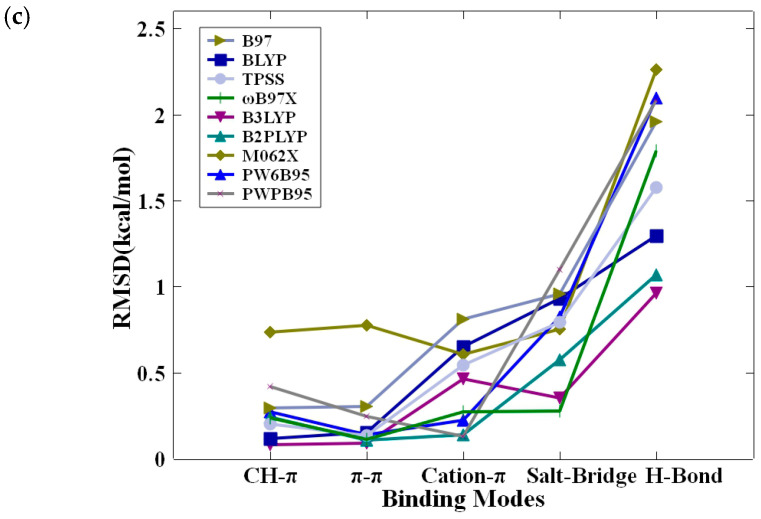
RMSD of calculated intermolecular interaction energies (in kcal/mol) for the nine DFT methods against those of CCSD(T)/CBS as a function of modes of nonbonded interactions. (**a**) DFT/def2-SVP, (**b**) DFT/def2-TZVP, (**c**) DFT/def2-QZVP.

Figure 3 provides a more detailed and distributed analysis of the root-mean-square deviation (RMSD) in calculated interaction energies as a function of modes of nonbonded interactions across nine different DFT methods, each assessed at three distinct basis set levels: def2-SVP, def2-TZVP, and def2-QZVP. Overall, the performance of all nine DFT methods, as judged from the RMSD values, are generally much better for nonbonded π-interactions (CH–π, π–π stacking, and cation–π interactions) than the polar and charged interactions (hydrogen bonding and salt bridge interactions). Notably, Figure 3a unveils an unexpected observation: a remarkably high RMSD for salt bridge interactions when using methods like B3LYP, BLYP, PW6B95, PW6B95 RIJCOSX, RI B97, RI TPSS, and ωB97X, coupled with the def2-SVP basis set. Intriguingly, for the def2-TZVP and def2-QZVP basis sets, the highest RMSD is associated with hydrogen bond interactions. A careful examination of detailed interaction energies as listed in Table 2 and Tables S1–S3 for hydrogen bonded motifs uncovers two important characteristics of the errors. First, the computed nonbonded interaction energy from all DFT approaches is significantly less negative than the very attractive (negative) values obtained using the CCSD(T)/CBS method. Second, there appears to be a consistent bias (positive difference between DFT and CCSD(T)) across all DFT techniques, as indicated by the strong resemblance between the magnitudes of the mean unsigned error (MUE) and average error (AVG) and the root-mean-square deviation (RMSD).

**Figure 4:**
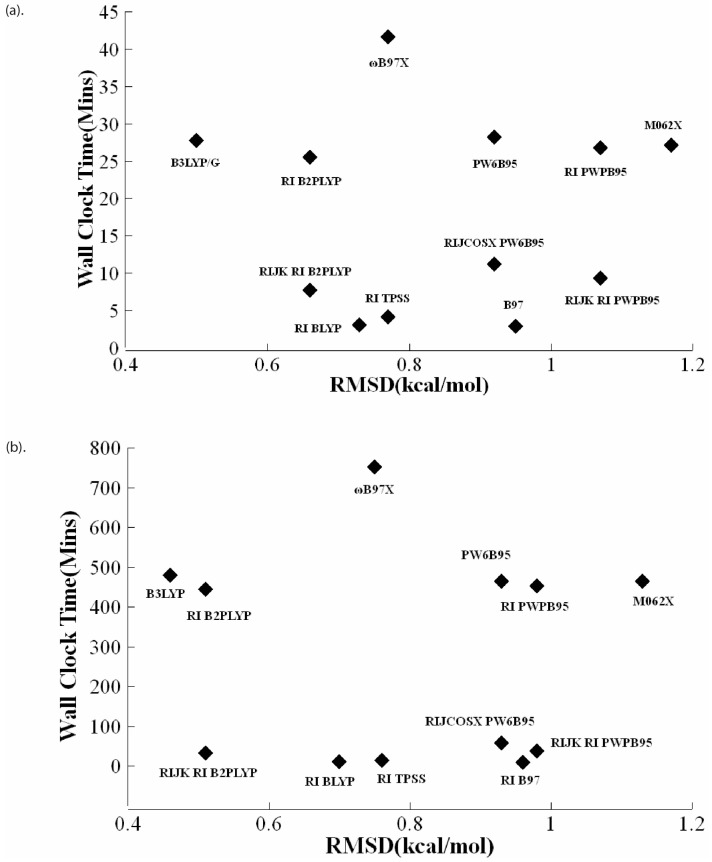
Performance of nine DFT methods and their variants as applied to the 49 binding motifs of PKIs in protein kinases. The average RMSD in interaction energies is calculated with respect to the CCSD(T)/CBS method. For each DFT method, the calculation times represent the averaged time over all 49 motifs. (**a**) DFT/def2-TZVP and (**b**) DFT/def2-QZVP.

Another dimension of consideration, particularly relevant for large biological systems, is the computational cost associated with DFT calculations. A delicate trade-off exists between methodological accuracy and computational efficiency. The accuracy can be quantified by RMSD values relative to the CCSD(T)/CBS benchmarks. The computational cost is measured by CPU time. For equal configuration of computing hardware, all DFT calculations were carried out on Dell workstations equipped with 2.7 GHz Intel Core i7 6820HQ CPU and 32 GB core memory. The ORCA program was executed in parallel on four processors using the PAL4 command. CPU times are calculated as the average over the entire set of 49 motifs of nonbonded interactions. Figure 4 presents a comparative analysis of various DFT methods, evaluating them on the dual criteria of accuracy and computational cost. The plot maps accuracy against CPU time (in min) for various implementations of the nine exchange-correlation functionals (see Table 2) coupled to def2-TZVP and def2-QZVP basis sets. Lower RMSD values signify greater accuracy and short CPU times indicate less computational expense. Consequently, the optimal DFT method would ideally be situated in the lower-left quadrant of the plot, representing both high accuracy and low computational cost. Based on these considerations, the B3LYP/def2-TZVP combination, with an RMSD of 0.50 kcal/mol and an average CPU time of 27.7 min, aligns closest with the criteria of achieving the lowest RMSD while also requiring the least CPU time, making it the top performing choice under the metrics considered. For applications that require the highest accuracy, B3LYP/def2-QZVP offers the best RMSD value of 0.46 kcal/mol, but with an extremely high average CPU cost of 479.8 min. The next best performing combination is RIJK RI-B2PLYP/def2-QZVP with an RMSD of 0.51 kcal/mol and an averaged CPU time of 33.2 min. For applications that can tolerate a slight loss of accuracy, the RIJK RI-B2PLYP/def2-TZVP combination (with a RMSD 0.66 kcal/mol) has the advantage of consuming nearly one fourth of the CPU time (7.7/33.2) required by RIJK RI-B2PLYP/def2-QZVP.

As pointed out earlier, for functionals with various numerical algorithms of implementation, i.e., RI, RIJCOSX, and RI RIJK, there was no noticeable difference in accuracy. Remarkably, large differences in CPU time were observed. For example, the averaged CPU time for RIJK RI B2PLYP/def2-QZVP and RI B2PLYP/def2-QZVP are 33.2 min and 444.2 min, respectively. This represents a CPU time reduction of 13.4 fold, which can be a favorable factor of consideration for large biological systems like the PKI-bound protein kinases.

## 3. Theory and Methods

### Quantum Chemical Calculation of Intermolecular Interaction Energies

**Gas phase intermolecular interaction energy.** The energies of intermolecular interactions in the gas phase were calculated by means of the supramolecular approach. In the supramolecular approach, the energy of interaction between molecules *A* and *B* is defined as the difference between the energy of the interacting dimer and the energies of the monomers:

(1) $${∆E}_{int}^{g}=E_{AB}-E_{A}-E_{B}$$

Nine DFT methods are employed to calculate the gas phase intermolecular interaction energies according to Equation (1) for benchmarking purpose: BLYP [19,20], TPSS [21], B97 [22], ωB97X [23], B3LYP [24,25], M062X [26], PW6B95 [27], B2PLYP [28] and PWPB95 [29]. For each of the DFT methods, three basis sets are applied: def2-QZVP, def2-TZVP, and def2-SVP [31,73]. It is well known that traditional pure DFT functionals lack an account of dispersion interactions, and thus are inadequate for the treatment of dispersion force dominated nonbonded interactions such as CH–π interactions and π–π stacking interactions. To overcome this problem, the D3 level dispersion correction developed by Grimme [30] is applied. Specifically, the atom-pairwise dispersion correction with the Becke–Johnson damping scheme (D3BJ) [80] is adopted. Here, the dispersion correction is calculated as follows by adding over all atom pairs in the systems:

(2) $$E_{\mathrm{disp}}^{D3(BJ)}=-\frac{1}{2}\sum_{A\neq B}s_{6}\frac{C_{6}^{\mathrm{AB}}}{R_{\mathrm{AB}}^{6}+{\left[f(R_{\mathrm{AB}}^{0})\right]}^{6}}+s_{8}\frac{C_{8}^{\mathrm{AB}}}{R_{\mathrm{AB}}^{8}+{\left[f(R_{\mathrm{AB}}^{0})\right]}^{8}}\;$$

where $C_{6}^{AB}$ and $C_{8}^{AB}$ are the averaged dispersion coefficients for the interacting atoms A and B at sixth and eighth order. $R_{AB}\;$ represents internuclear distance between atom A and atom B and $S_{n}$ (n = 6, 8) are global scaling factors that are used to adjust the correction to the repulsive behavior of the used DFT method [81].

In practical calculations, the coordinates of all atoms are taken directly from X-ray crystal structures of the PKI-bound protein kinases. The positions of hydrogen atoms are patched through geometry optimization calculations at the HF/6-31+G level using the Gaussian 09 program [64]. All the DFT calculations are carried out using the ORCA 04 [33] program. Last but not least, the basis set superposition error (BSSE) was corrected by the Boys and Bernardi Counter Poise Method [79].

**The CCSD(T)/CBS Method.** To establish a benchmarking reference, the CCSD(T) method at the complete basis set is employed to calculate the gas phase intermolecular interaction energy according to Equation (1). As mentioned in Section 2.1, the CCSD(T)/CBS Method is widely regarded as the gold standard for treatment of intermolecular interaction energies. Due to the high computational cost of CCSD(T) calculation, the complete basis set (CBS) limit is achieved by means of an extrapolation scheme [14]. In that scheme, the CCSD(T)/CBS level interaction energy is given by:

(3) $$∆E_{\mathrm{CBS}}^{\mathrm{CCSD}(T)}=∆E_{\mathrm{CBS}}^{\mathrm{MP}2}+(∆E^{\mathrm{CCSD}(T)}-∆E^{\mathrm{MP}2}){|}_{\mathrm{small}\;\mathrm{basis}\;\mathrm{set}}$$

where ${∆E}_{CBS}^{MP2}$ is the MP2 level interaction energy with complete basis set. It is calculated using the two-point basis set extrapolation scheme developed by Halkier and co-workers [66,67]:

(4) $$E_{corr,\lim}=\frac{X^{3}}{X^{3}-{\left(X-1\right)}^{3}}E_{corr,X}-\frac{{\left(X-1\right)}^{3}}{X^{3}-{\left(X-1\right)}^{3}}E_{corr,X-1}$$

where $E_{corr,X}$ is the correlation energy obtained with the correlation consistent basis set (cc-pVXZ) with cardinal number (X = T and Q) and $E_{corr,\lim}$ is the basis set limit value of the correlation energy. The post-MP2 correction $\left(∆E^{\mathrm{CCSD}(T)}-∆E^{\mathrm{MP}2}\right)$ , also known as “coupled cluster correction”, is calculated using the augmented correlation consistent basis set aug-cc-pVDZ.

**Solution phase intermolecular interaction energy.** The energy of intermolecular interaction in the solution phase was evaluated indirectly by means of a thermodynamic scheme. For a detailed description of the scheme, interested readers can refer to Reference [6]. According to the scheme, the binding energy for complex formation in the solution phase can be evaluated indirectly by calculating intermolecular interaction energies in the gas phase, ${∆E}_{int\;}^{g}$ , followed by a correction for the dehydration energy ${∆E}_{Deh}$ :

(5) $${∆E}_{int\;}^{aq}={∆E}_{int\;}^{g}+{∆E}_{Deh}\;$$

The dehydration energy for the complex formation is defined by:

(6) $${∆E}_{Deh\;}={∆G}_{AB}^{sol}-∆G_{A}^{sol}-{∆G}_{B}^{sol}$$

where ${∆G}_{i}^{sol}$ , i = *AB*, *A*, *B* represents the free energies of solvation for the complex *AB*, and the monomers *A*, *B*, respectively. The SM5.42R Solvation Model of Cramer and Truhlar [69] as implemented in the 2008 R1 version of GAMESS [82] was adopted for the evaluation of those free energies of solvation. The SM5.42R model was chosen for its reported improvement of accuracy in predicting solvation energies [69]. In comparison to PCM and COSMO, it takes into account a wider range of solvent parameters, enabling a more complex and in-depth description of solvent–solute interactions.

## 4. Conclusions

Accurate quantification of the strengths of nonbonded interactions in large biological systems, like PKIs in their targeted proteins, is crucial for studying molecular recognition of ligands in proteins. However, high-accuracy computational methods often come at a substantial computational cost. This study navigates this trade-off by comprehensively assessing nine density functional theory (DFT) methods for their ability to balance accuracy and efficiency.

From a database of 2139 kinase–inhibitor crystal structures, we extracted a library of 49 diverse nonbonded interaction motifs, encompassing CH–π, π–π stacking, cation–π, hydrogen bonding, and salt bridge interactions. Using CCSD(T)/CBS as reference, we systematically benchmarked BLYP, TPSS, B97, ωB97X, B3LYP, M062X, PW6B95, B2PLYP, and PWPB95 functionals with D3BJ dispersion correction alongside def2-SVP, def2-TZVP, and def2-QZVP basis sets. RI, RI-JK, and RIJCOSX approximations were employed for specific functionals. The B3LYP/def2-TZVP combination emerges as the optimal choice, demonstrating a balanced performance with an RMSD of 0.50 kcal/mol and an average CPU time of 27.7 min. For applications demanding utmost accuracy, the B3LYP/def2-QZVP combination excels in RMSD (0.46 kcal/mol) but incurs a significantly higher average CPU cost of 479.8 min. The RIJK RI-B2PLYP/def2-QZVP combination provides a viable alternative, achieving a balance with an RMSD of 0.51 kcal/mol and an averaged CPU time of 33.2 min. Notably, the RIJK RI-B2PLYP/def2-TZVP combination, while slightly less accurate (RMSD 0.66 kcal/mol), presents a substantial reduction in CPU time (7.7/33.2) against RIJK RI-B2PLYP/def2-QZVP, making it advantageous for applications that can tolerate a moderate loss of accuracy. Remarkably, different numerical implementations of the same functional can drastically impact CPU time, with RIJK RI B2PLYP/def2-QZVP requiring 13.4 times less time than RI B2PLYP/def2-QZVP.

These findings provide valuable insights for choosing the optimal DFT method for studying nonbonded interactions in large biological systems, particularly when computational expense is a major concern. This work paves the way for robust, high-throughput structure-based drug design targeting protein kinases through accurate modeling of critical nonbonded interactions.

Moreover, the calculated CCSD(T) energies extend beyond this study, offering a valuable benchmark for future testing of methods for calculating intermolecular interactions. Furthermore, the established library of 3D motifs stands as a significant contribution itself, providing a valuable resource for realistic molecular modeling of ligand binding with protein in general. 

## Figures and Tables

**Table 1:** List of motifs of nonbonded interaction pairs between PKIs and their interacting residues ^a^.

No.	Mode of Interaction	PDB ID	Intermolecular Pair	Angle	Distance (Å) ^b^			Ref.
1	CH–π	1H1Q	2A6…A31		3.32			[37]
2		4BDB	ODO…L354	-	3.93			[38]
3		2BTS	U32…I10	-	3.55			[39]
4		2XIY	XIY…L174	-	3.57			[40]
5		1UU9	BI3…L88	-	3.81			[41]
6		2IO6	330…I374	-	4.41			[42]
7		4GUE	QCT…L147	-	3.61			[43]
8		3OOG	YTP…A70	-	3.34			-
9		4FKO	20K…I10	-	3.72			-
10		3QL8	X01…L134	-	3.23			-
11		4RC2	3O8…L174	-	3.43			-
12		4RC3	3OA…A56	-	3.27			-
13		3QX4	X4B…L134	-	3.61			-
14	π–π stacking	2V7O	DRN…F90	89.64 ^c^	3.77			[44]
15		3I4B	Z48…Y134	6.8	4.31			[45]
16		4DGO	0JW…F113	43.85	3.55			-
17		3QQF	X07…F82	70.14	3.76			-
18		3B2T	M33…Y566	10.09	3.83			[46]
19		3R6X	X84…F82	74.54	3.71			-
20		4WG4	UWB…Y131	5.57	4.08			-
21		1PXN	CK6…F82	16.8	3.62			[47]
22		3ROY	22Z…F82	68.31	3.75			-
23		2C6O	4SP…F82	20.3	4.01			[48]
24		3RAK	03Z…F82	41.29	3.81			[49]
25		4EL9	AFE…F79	19.17	3.48			[50]
26	Cation–π	1PXL	CK4…K33	-	4.62			[51]
27		1FGI	SU1…K514	-	5.15			[52]
28		1M2Q	MNX…K68	-	4.21			[53]
29		2VGP	AD6…K180	-	5.06			[54]
30		3OWP	2SB…K72	-	3.59			-
31		3SQQ	99Z…K33	-	5.12			[49]
32		3RJC	06Z…K33	-	4.77			[49]
33		3QTW	X3A…K89	-	3.51			[49]
34	H-Bond ^d^	3SW4	18K…L83	158.59	166.51	2.88	2.94	-
35		3ZLY	YSO…M146	153.12	164.48	2.97	3.26	[55]
36		3RZB	02Z…L83	143.48	162.20	2.96	3.06	[49]
37		3RPY	27Z…L83	141.82	167.13	2.89	3.24	[49]
38		3R28	XA0…L83	155.87	134.34	2.88	2.98	-
39		3QZI	X72…L83	132.02	148.51	2.98	2.98	-
40		3SXF	BK5…Y131	161.45		3.14		
41		2R3P	3SC…L83	141.41	168.79	2.82	3.35	[56]
42	Salt bridge	3MA3	01I…K67	-	3.38			[57]
43		4L9I	8PR…D271	-	3.96			[58]
44		1NVQ	UCN…E91	-	3.48			[59]
45		4JIK	1KO…D148	-	3.22			[60]
46		1BX6	BA1…E127	-	5.05			[61]
47		4WG4	UWB…E135	-	4.16			-
48		4MTA	2D2…K67	-	3.67			[62]
49		1E9H	INR…K33	-	3.65			[63]

^a^: All ligands (designated by the three-letter ligand IDs) and their interacting residues are extracted from chain A of the corresponding PDB files. ^b^: The closest distance between nonhydrogen atoms (C, O, or N) of the PKI and those of its interacting residue. ^c^: Angle (in degree) for π–π stacking motifs is measured between the two π planes. ^d^: All motifs of H-bonding interactions contain dual H-bonding except motif 40. Thus, two sets of parameters are given.

**Table 2:** Intermolecular interaction energies (in kcal/mol) calculated at the CCSD(T)/CBS level for the 3D binding motifs of protein kinase inhibitors in protein kinases.

No.	Mode of Interaction	PDB ID	Intermolecular Pair	${∆E}_{CCSD(T)}^{g}$ (kcal/mol)	ΔE_Deh_ (kcal/mol)	${∆E}_{int}^{aq}$ (kcal/mol) ^a^
1	CH–π	1H1Q	2A6…A31	−1.9	0.5	−1.4
2		4BDB	ODO…L354	−2.2	0.4	−1.8
3		2BTS	U32…I10	−2.4	0.8	−1.6
4		2XIY	XIY…L174	−2.3	0.3	−2.0
5		1UU9	BI3…L88	−3.6	0.3	−3.3
6		2IO6	330…I374	−0.7	0.0	−0.7
7		4GUE	QCT…L147	−3.4	0.7	−2.7
8		3OOG	YTP…A70	−1.9	0.7	−1.2
9		4FKO	20K…I10	−2.3	0.0	−2.3
10		3QL8	X01…L134	−2.5	0.0	−2.5
11		4RC2	3O8…L174	−2.1	0.2	−1.9
12		4RC3	3OA…A56	−1.0	−1.0	−2.0
13		3QX4	X4B…L134	−1.0	0.1	−0.9
14	π–π Stacking	2V7O	DRN…F90	−1.3	0.0	−1.3
15		3I4B	Z48…Y134	−1.0	0.3	−0.7
16		4DGO	0JW…F113	−4.4	1.6	−2.8
17		3QQF	X07…F82	−2.4	0.7	−1.7
18		3B2T	M33…Y566	−2.0	0.5	−1.5
19		3R6X	X84…F82	−1.9	0.4	−1.5
20		4WG4	UWB…Y131	−0.6	0.3	−0.3
21		1PXN	CK6…F82	−0.7	0.6	−0.1
22		3ROY	22Z…F82	−2.4	0.6	−1.8
23		2C6O	4SP…F82	−1.1	0.8	−0.3
24		3RAK	03Z…F82	−1.7	0.1	−1.6
25		4EL9	AFE…F79	−1.9	0.4	−1.5
26	Cation–π	1PXL	CK4…K33	−6.5	5.3	−1.2
27		1FGI	SU1…K514	−4.4	2.5	−1.9
28		1M2Q	MNX…K68	−2.0	−0.3	−2.3
29		2VGP	AD6…K180	−1.5	1.0	−0.5
30		3OWP	2SB…K72	−9.2	4.5	−4.7
31		3SQQ	99Z…K33	−2.1	0.8	−1.3
32		3RJC	06Z…K33	−2.1	0.5	−1.6
33		3QTW	X3A…K89	−8.4	8.6	0.2
34	H-Bond	3SW4	18K…L83	−6.0	4.8	−1.2
35		3ZLY	YSO…M146	−4.9	3.7	−1.2
36		3RZB	02Z…L83	−7.0	5.2	−1.8
37		3RPY	27Z…L83	−7.0	4.4	−2.6
38		3R28	XA0…L83	−4.8	4.0	−0.8
39		3QZI	X72…L83	−4.6	0.6	−4.0
40		3SXF	BK5…Y131	−3.8	3.1	−0.7
41		2R3P	3SC…L83	−6.1	3.3	−2.8
42	Salt bridge	3MA3	01I…K67	−111.0	102.6	−8.4
43		4L9I	8PR…D271	−100.3	94.5	−5.8
44		1NVQ	UCN…E91	−100.0	98.6	−1.4
45		4JIK	1KO…D148	−115.9	112.2	−3.7
46		1BX6	BA1…E127	−68.2	67.5	−0.7
47		4WG4	UWB…E135	−92.6	87.5	−5.1
48		4MTA	2D2…K67	−106.6	99.4	−7.2
49		1E9H	INR…K33	−101.5	98.5	−3.0

^a^ The solution phase interaction energy was calculated via ${∆E}_{int\;}^{aq}={∆E}_{CCSD(T)\;}^{g}+{∆E}_{Deh}$ according to Equation (5).

**Table 3:** List of DFT methods.

Functional	Implementation ^a^	HF Exchange ^b^ (%)	Type ^c^	Ex. Functional Corr Functional	Ref.
BLYP	RI BLYP	0	GGA	Becke 1988 Lee-Yang-Parr 1988	[19,20]
TPSS	RI TPSS	0	Meta-GGA	TPSS TPSS	[21,74]
B97	RI B97	26.93	hybrid GGA	B97-2 B97-3	[29,75]
ωB97X	ωB97X	15.77	range-separated hybrid-GGA	LRC hybrid functionals	[23]
B3LYP	B3LYP	20	hybrid GGA	Becke 1988 Lee-Yang-Parr 1988	[19,20]
M062X	M062X	54	Meta-hybrid GGA	M06-2X M06-2X	[26]
PW6B95	PW6B95	28	Meta-hybrid GGA	PW6B95 PW6B95	[27]
	RIJCOSX PW6B95				
B2PLYP	RI B2PLYP	54	Double-Hybrid-GGA	Becke 1988	[19,20]
	RIJK RI B2PLYP			Lee-Yang-Parr 1988	[28]
PWPB95	RI PWPB95	50	Double-Hybrid-Meta GGA	Perdew–Wang32	[76,77]
	RIJK RI PWPB95			Becke9533	[29,78]

^a^: RI denotes the Resolution of Identity (RI) approximation [70]. RIJCOSX is an approximation that uses density fitting for the Coulomb (J) integrals and numerical chain-of-sphere integration for the HF Exchange integrals (COSX) [71]. “RI RIJK” stands for resolution of identity (RI) approximation for the perturbation step and RIJK (RI approximation is applied to both Coulomb and HF Exchange integrals) [72] for the HF-SCF step. ^b^: the percentage of HF exchange in the functional. ^c^: GGA denotes generalized gradient approximation.

**Table 4:** Errors of the studied methods for 49 motifs with respect to the benchmark (CCSD(T)/CBS) calculations.

DFT Method	RMSD (kcal/mol)			MAE (kcal/mol)			AVG (kcal/mol)			MAX%		
	def2-QZVP	def2-TZVP	def2-SVP	def2-QZVP	def2-TZVP	def2-SVP	def2-QZVP	def2-TZVP	def2-SVP	def2-QZVP	def2-TZVP	def2-SVP
BLYP	0.70	0.73	1.25	0.48	0.48	0.76	0.15	0.09	−0.28	33.2	34.0	38.1
TPSS	0.76	0.77	0.97	0.52	0.51	0.64	0.23	0.21	−0.09	39.7	41.2	38.5
B97	0.96	0.95	1.11	0.70	0.66	0.77	0.14	0.11	−0.18	50.4	51.3	51.0
ωB97X	0.75	0.77	0.88	0.43	0.43	0.55	0.23	0.20	0.01	43.1	43.5	39.8
B3LYP	0.46	0.50	0.96	0.29	0.32	0.55	0.08	0.05	−0.24	25.9	26.6	26.6
M062X	1.13	1.17	1.14	0.93	0.94	1.00	0.93	0.93	0.82	103.1	106.4	118.6
PW6B95	0.93	0.92	0.90	0.57	0.54	0.62	0.50	0.47	0.23	46.1	47.6	54.4
B2PLYP	0.51	0.66	0.93	0.35	0.45	0.75	0.32	0.45	0.50	26.9	43.4	76.3
PWPB95	0.98	1.07	1.05	0.68	0.74	0.79	0.67	0.74	0.65	51.7	61.9	86.5

RMSD: root mean square deviation. MAE: mean absolute error. AVG: average signed error. MAX%: the largest percentage error in the set relative to the CCSD(T)/CBS interaction energy.

## Data Availability

Coordinates of 49 motifs of nonbonded interactions between PKIs and their respective binding protein kinases are available from https://rocketsutoledo-my.sharepoint.com/:f:/g/personal/xhu_rockets_utoledo_edu/EjY6HV9di7BBmZVh_MVoyUUBLJIKBHyDskH3GjBAzd-80A?e=aeKLVn. Access to the data set needs to be authorized by the authors upon request. The data are not publicly available due to internal campus network security protocol.

## Supplementary Materials

The following supporting information can be downloaded at: www.mdpi.com/xxx/s1, Table S1: Gas phase intermolecular interaction energies at the DFT/def2-SVP level for the 49 binding motifs of PKIs in protein kinases. Table S2: Gas phase intermolecular interaction energies at the DFT/def2-TZVP level for the 49 binding motifs. Table S3: Gas phase intermolecular interaction energies (in kcal/mol) at the DFT/def2-QZVP level for the 49 binding motifs.
