# Supplementary material for: An Assessment of Dispersion-Corrected DFT Methods for Modeling Nonbonded Interactions in Protein Kinase Inhibitor Complexes
Source: Molecules. 2024 Jan 6;29(2):0. doi: 10.3390/molecules29020304 (PMC11154270; doi:10.3390/molecules29020304)
Supplement: Supplementary file 1 [file molecules-29-00304-s001.zip › molecules-2719919-supplementary.pdf]

Table S1. Gas phase intermolecular interaction energies (in kcal/mol) at the DFT/Def2-SVP level for the 49 binding motifs of PKIs in protein kinases.

No.	Interaction	PDB ID	CCSD(T)/CBS	DFT/Def2-SVP									
				BLYP	TPSS	B97	ωB97X	B3LYP	M062X	PW6B95	B2PLYP	PWPB95
1	CH-π	1H1Q	-1.9	-1.7	-1.5	-1.8	-1.7	-1.7	-1.4	-1.5	-1.1	-1.1
2		4BDB	-2.2	-2.2	-2.4	-2.5	-2.5	-2.2	-1.3	-2.3	-1.7	-1.9
3		2BTS	-2.4	-2.3	-2.2	-2.5	-2.5	-2.4	-1.3	-2.0	-1.5	-1.5
4		2XIY	-2.3	-2.1	-2.1	-2.4	-2.3	-2.1	-1.5	-2.2	-1.4	-1.7
5		1UU9	-3.6	-3.7	-3.5	-4.0	-3.9	-3.7	-2.2	-3.1	-2.7	-2.7
6		2IO6	-0.7	-0.7	-0.9	-0.9	-0.7	-0.7	-0.1	-0.7	-0.5	-0.5
7		4GUE	-3.4	-3.4	-3.1	-3.5	-3.6	-3.4	-2.4	-3.1	-2.3	-2.5
8		3OOG	-1.9	-1.8	-1.6	-1.9	-1.8	-1.8	-1.3	-1.6	-1.1	-1.1
9		4FKO	-2.3	-2.5	-2.5	-2.8	-2.6	-2.5	-1.0	-2.0	-1.7	-1.5
10		3QL8	-2.5	-2.5	-2.3	-2.8	-2.5	-2.5	-1.7	-2.2	-1.3	-1.6
11		4RC2	-2.1	-2.0	-1.9	-2.4	-2.2	-2.1	-1.2	-1.5	-1.2	-1.1
12		4RC3	-1.0	-0.9	-0.8	-1.1	-0.9	-0.9	-0.4	-0.4	-0.2	-0.1
13		3QX4	-1.0	-0.9	-1.0	-1.2	-1.0	-1.0	-0.4	-0.9	-0.5	-0.6
14	π -π	2V7O	-1.3	-1.5	-1.4	-1.6	-1.3	-1.4	-0.2	-1.1	-1.0	-0.9
15		3I4B	-1.0	-1.0	-1.2	-1.3	-1.1	-1.0	-0.4	-1.1	-0.8	-0.9
16		4DGO	-4.4	-4.5	-4.3	-4.8	-4.5	-4.5	-3.4	-4.4	-3.5	-3.8
17		3QQF	-2.4	-2.2	-2.2	-2.4	-2.1	-2.3	-1.6	-2.1	-1.9	-1.8
18		3B2T	-2.0	-1.7	-1.9	-2.0	-1.7	-1.8	-0.8	-1.7	-1.4	-1.4
19		3R6X	-1.9	-1.9	-1.9	-2.2	-1.7	-2.0	-0.9	-1.7	-1.5	-1.4
20		4WG4	-0.6	-0.6	-0.7	-0.8	-0.5	-0.5	0.0	-0.6	-0.4	-0.4
21		1PXN	-0.7	-0.7	-0.8	-0.9	-0.6	-0.6	0.1	-0.6	-0.4	-0.4
22		3ROY	-2.4	-2.2	-2.2	-2.4	-2.2	-2.3	-1.6	-2.1	-1.9	-1.9
23		2C6O	-1.1	-1.0	-1.1	-1.2	-1.0	-0.9	-0.3	-1.0	-0.7	-0.8
24		3RAK	-1.7	-1.6	-1.7	-1.8	-1.6	-1.6	-0.8	-1.4	-1.3	-1.2
25		4EL9	-1.9	-2.0	-1.9	-2.3	-2.0	-1.8	-1.0	-1.6	-1.0	-1.2

(Continued)

Table S1 *(Continued)*

No.	Interaction	PDB ID	CCSD(T) /CBS	DFT/Def2-SVP								
				BLYP	TPSS	B97	ωB97X	B3LYP	M062X	PW6B95	B2PLYP	PWPB95
26	Cation-π	1PXL	-6.5	-6.2	-6.2	-6.3	-6.3	-6.3	-5.9	-6.3	-6.2	-6.2
27		1FGI	-4.4	-4.9	-4.9	-5.0	-4.4	-4.7	-3.6	-4.5	-4.3	-4.2
28		1M2Q	-2.0	-2.8	-2.6	-2.8	-2.0	-2.4	-0.7	-2.0	-1.8	-1.6
29		2VGP	-1.5	-1.5	-1.5	-1.5	-1.4	-1.4	-1.1	-1.4	-1.3	-1.3
30		3OWP	-9.2	-9.8	-9.6	-10.1	-9.5	-9.7	-8.3	-9.4	-8.7	-8.8
31		3SQQ	-2.1	-2.4	-2.4	-2.5	-2.1	-2.3	-1.1	-2.0	-1.8	-1.6
32		3RJC	-2.1	-2.4	-2.3	-2.5	-2.0	-2.2	-1.2	-2.0	-1.8	-1.7
33		3QTW	-8.4	-10.4	-9.6	-10.2	-8.3	-9.7	-7.8	-8.9	-8.7	-8.3
34	H-Bonding	3SW4	-6.0	-4.7	-4.7	-4.1	-4.5	-5.4	-3.5	-3.9	-4.0	-3.3
35		3ZLY	-4.9	-4.2	-4.2	-3.9	-4.0	-4.6	-3.0	-3.6	-3.8	-3.2
36		3RZB	-7.0	-6.1	-5.9	-5.6	-5.8	-6.5	-4.9	-5.2	-5.3	-4.7
37		3RPY	-7.0	-5.8	-5.6	-5.2	-5.7	-6.4	-5.1	-5.3	-5.4	-4.9
38		3R28	-4.8	-3.1	-3.0	-2.4	-2.9	-3.6	-2.7	-2.8	-2.8	-2.3
39		3QZI	-4.6	-3.5	-3.3	-2.8	-3.3	-3.9	-3.1	-3.1	-3.1	-2.7
40		3SXF	-3.8	-2.7	-2.7	-2.2	-2.5	-3.1	-2.0	-2.2	-2.3	-1.8
41		2R3P	-6.1	-4.5	-4.2	-3.8	-4.3	-4.9	-3.8	-4.0	-4.1	-3.6
42	Salt bridge	3MA3	-111.0	-113.5	-112.9	-112.7	-112.8	-113.4	-112.1	-112.7	-112.0	-111.9
43		4L9I	-100.3	-103.8	-103.2	-103.0	-102.3	-103.1	-100.8	-102.0	-101.8	-101.2
44		1NVQ	-100.0	-101.2	-100.9	-100.5	-101.2	-101.5	-100.3	-100.6	-100.1	-99.8
45		4JIK	-115.9	-118.7	-118.3	-118.2	-118.4	-118.9	-117.2	-117.5	-117.4	-116.6
46		1BX6	-68.2	-72.2	-70.1	-70.7	-68.4	-68.8	-68.0	-68.7	-68.3	-68.3
47		4WG4	-92.6	-95.1	-93.9	-94.1	-93.0	-94.0	-92.2	-93.1	-92.8	-92.4
48		4MTA	-106.6	-108.9	-108.5	-108.2	-108.6	-109.0	-106.7	-107.7	-107.6	-106.9
49		1E9H	-101.5	-103.2	-102.1	-102.1	-102.7	-103.5	-102.6	-102.6	-102.1	-101.9

Table S2. Gas phase intermolecular interaction energies (in kcal/mol) at the DFT/Def2-TZVP level for the 49 binding motifs of PKIs in protein kinases.

No.	Interaction	PDB ID	CCSD(T) /CBS	DFT/Def2-TZVP								
				BLYP	TPSS	B97	ωB97X	B3LYP	M062X	PW6B95	B2PLYP	PWPB95
1	CH-π	1H1Q	-1.9	-1.6	-1.5	-1.8	-1.8	-1.7	-1.4	-1.6	-1.4	-1.4
2		4BDB	-2.2	-2.1	-2.4	-2.5	-2.6	-2.2	-1.4	-2.4	-2.0	-2.1
3		2BTS	-2.4	-2.3	-2.2	-2.5	-2.6	-2.4	-1.5	-2.1	-1.9	-1.8
4		2XIY	-2.3	-2.0	-2.1	-2.4	-2.5	-2.1	-1.6	-2.2	-1.8	-1.9
5		1UU9	-3.6	-3.7	-3.6	-4.0	-4.0	-3.7	-2.4	-3.2	-3.2	-2.9
6		2IO6	-0.7	-0.7	-0.8	-0.9	-0.7	-0.7	-0.1	-0.7	-0.6	-0.5
7		4GUE	-3.4	-3.3	-3.1	-3.5	-3.8	-3.4	-2.6	-3.2	-2.8	-2.8
8		3OOG	-1.9	-1.7	-1.6	-1.9	-2.0	-1.9	-1.5	-1.7	-1.5	-1.4
9		4FKO	-2.3	-2.5	-2.5	-2.9	-2.7	-2.5	-1.1	-2.0	-2.0	-1.7
10		3QL8	-2.5	-2.4	-2.2	-2.8	-2.7	-2.5	-1.9	-2.3	-1.9	-1.9
11		4RC2	-2.1	-2.0	-2.0	-2.4	-2.4	-2.1	-1.4	-1.6	-1.7	-1.4
12		4RC3	-1.0	-0.8	-0.8	-1.1	-1.0	-0.9	-0.6	-0.6	-0.6	-0.4
13		3QX4	-1.0	-0.9	-1.0	-1.2	-1.1	-1.0	-0.5	-0.9	-0.7	-0.7
14	π -π	2V7O	-1.3	-1.4	-1.4	-1.6	-1.3	-1.4	-0.2	-1.1	-1.1	-0.9
15		3I4B	-1.0	-1.0	-1.3	-1.3	-1.2	-1.0	-0.5	-1.2	-0.9	-1.0
16		4DGO	-4.4	-4.5	-4.4	-4.9	-4.7	-4.6	-3.6	-4.4	-4.1	-4.1
17		3QQF	-2.4	-2.2	-2.3	-2.5	-2.3	-2.4	-1.8	-2.2	-2.2	-2.1
18		3B2T	-2.0	-1.8	-2.0	-2.1	-1.9	-1.9	-1.0	-1.8	-1.7	-1.6
19		3R6X	-1.9	-1.9	-1.9	-2.2	-1.8	-2.0	-1.0	-1.7	-1.7	-1.5
20		4WG4	-0.6	-0.5	-0.7	-0.7	-0.6	-0.5	0.0	-0.6	-0.5	-0.5
21		1PXN	-0.7	-0.6	-0.8	-0.9	-0.7	-0.7	0.0	-0.6	-0.6	-0.5
22		3ROY	-2.4	-2.2	-2.3	-2.5	-2.3	-2.4	-1.8	-2.2	-2.2	-2.1
23		2C6O	-1.1	-0.9	-1.2	-1.2	-1.1	-1.0	-0.4	-1.1	-0.9	-0.9
24		3RAK	-1.7	-1.6	-1.7	-1.9	-1.7	-1.7	-0.9	-1.5	-1.5	-1.3
25		4EL9	-1.9	-2.1	-2.0	-2.4	-2.0	-2.0	-1.0	-1.6	-1.6	-1.4
26	Cation-π	1PXL	-6.5	-6.5	-6.5	-6.6	-6.6	-6.6	-6.2	-6.6	-6.5	-6.5
27		1FGI	-4.4	-4.8	-4.9	-5.0	-4.5	-4.7	-3.7	-4.6	-4.4	-4.3
28		1M2Q	-2.0	-2.7	-2.7	-2.9	-2.2	-2.4	-1.0	-2.2	-2.0	-1.8

(Continued)

Table S2 *(Continued)*

No.	Interaction	PDB ID	CCSD(T) /CBS	DFT/Def2-TZVP								
				BLYP	TPSS	B97	ωB97X	B3LYP	M062X	PW6B95	B2PLYP	PWPB95
29	Cation-π	2VGP	-1.5	-1.6	-1.6	-1.7	-1.5	-1.5	-1.3	-1.6	-1.5	-1.5
30		3OWP	-9.2	-9.9	-9.8	-10.3	-9.8	-9.9	-8.6	-9.6	-9.2	-9.1
31		3SQQ	-2.1	-2.3	-2.4	-2.5	-2.2	-2.2	-1.2	-2.0	-1.9	-1.7
32		3RJC	-2.1	-2.3	-2.4	-2.6	-2.1	-2.2	-1.4	-2.1	-2.0	-1.9
33		3QTW	-8.4	-9.8	-9.3	-9.9	-8.3	-9.3	-7.8	-8.7	-8.5	-8.2
34	H-Bonding	3SW4	-6.0	-4.5	-4.2	-3.8	-3.9	-5.0	-2.9	-3.3	-4.3	-3.1
35		3ZLY	-4.9	-3.9	-3.8	-3.5	-3.4	-4.2	-2.6	-3.1	-3.8	-2.9
36		3RZB	-7.0	-6.1	-5.6	-5.5	-5.4	-6.2	-4.5	-4.8	-5.6	-4.6
37		3RPY	-7.0	-5.6	-5.2	-4.9	-5.1	-6.0	-4.5	-4.8	-5.5	-4.6
38		3R28	-4.8	-3.2	-2.8	-2.4	-2.7	-3.6	-2.6	-2.6	-3.2	-2.4
39		3QZI	-4.6	-3.5	-3.2	-2.8	-3.1	-3.9	-3.0	-3.0	-3.5	-2.8
40		3SXF	-3.8	-2.7	-2.5	-2.2	-2.3	-3.0	-1.9	-2.0	-2.7	-1.8
41		2R3P	-6.1	-4.2	-3.8	-3.5	-3.8	-4.6	-3.4	-3.6	-4.3	-3.5
42	Salt bridge	3MA3	-111.0	-111.0	-110.5	-110.5	-110.9	-111.2	-110.7	-110.6	-110.4	-110.1
43		4L9I	-100.3	-101.6	-101.2	-101.0	-100.6	-101.2	-99.6	-100.2	-100.3	-99.6
44		1NVQ	-100.0	-99.4	-99.3	-98.9	-100.1	-99.9	-99.5	-99.2	-99.2	-98.8
45		4JIK	-115.9	-115.6	-115.5	-115.4	-116.1	-116.2	-115.4	-115.0	-115.4	-114.6
46		1BX6	-68.2	-70.1	-69.0	-69.4	-68.3	-68.3	-67.8	-68.3	-68.1	-68.1
47		4WG4	-92.6	-94.0	-93.2	-93.3	-92.8	-93.3	-92.4	-92.7	-92.6	-92.2
48		4MTA	-106.6	-107.1	-106.8	-106.6	-107.0	-107.4	-105.8	-106.1	-106.4	-105.6
49		1E9H	-101.5	-101.3	-100.6	-100.6	-101.4	-101.8	-101.6	-101.2	-100.9	-100.6

Table S3. Gas phase intermolecular interaction energies (in kcal/mol) at the DFT/Def2-QZVP level for the 49 binding motifs of PKIs in protein kinases

No.	Interaction	PDB ID	CCSD(T)/ CBS	DFT/Def2-QZVP								
				BLYP	TPSS	B97	ωB97X	B3LYP	M062X	PW6B95	B2PLYP	PWPB95
1	CH-π	1H1Q	-1.9	-1.7	-1.5	-1.8	-1.8	-1.8	-1.5	-1.6	-1.6	-1.5
2		4BDB	-2.2	-2.2	-2.4	-2.6	-2.6	-2.2	-1.4	-2.4	-2.1	-2.1
3		2BTS	-2.4	-2.3	-2.2	-2.6	-2.6	-2.4	-1.5	-2.1	-2.1	-1.8
4		2XIY	-2.3	-2.1	-2.1	-2.4	-2.5	-2.2	-1.6	-2.2	-2.0	-2.0
5		1UU9	-3.6	-3.7	-3.6	-4.1	-4.0	-3.7	-2.4	-3.2	-3.4	-3.0
6		2IO6	-0.7	-0.7	-0.8	-0.9	-0.7	-0.7	-0.1	-0.7	-0.6	-0.6
7		4GUE	-3.4	-3.3	-3.1	-3.6	-3.7	-3.4	-2.6	-3.2	-3.1	-2.9
8		3OOG	-1.9	-1.8	-1.6	-1.9	-2.0	-1.9	-1.5	-1.7	-1.6	-1.5
9		4FKO	-2.3	-2.5	-2.5	-2.9	-2.6	-2.5	-1.1	-2.0	-2.2	-1.8
10		3QL8	-2.5	-2.5	-2.3	-2.9	-2.7	-2.5	-1.9	-2.3	-2.2	-2.1
11		4RC2	-2.1	-2.0	-2.0	-2.5	-2.4	-2.1	-1.4	-1.6	-1.9	-1.5
12		4RC3	-1.0	-0.9	-0.8	-1.1	-1.0	-1.0	-0.6	-0.6	-0.7	-0.5
13		3QX4	-1.0	-0.9	-1.0	-1.2	-1.1	-1.0	-0.5	-0.9	-0.8	-0.8
14	π -π	2V7O	-1.3	-1.5	-1.5	-1.7	-1.3	-1.4	-0.3	-1.2	-1.3	-1.0
15		3I4B	-1.0	-1.0	-1.3	-1.3	-1.2	-1.0	-0.5	-1.2	-1.0	-1.0
16		4DGO	-4.4	-4.6	-4.4	-4.9	-4.7	-4.6	-3.6	-4.4	-4.3	-4.2
17		3QQF	-2.4	-2.2	-2.3	-2.5	-2.3	-2.4	-1.7	-2.2	-2.3	-2.1
18		3B2T	-2.0	-1.8	-2.0	-2.1	-1.9	-1.9	-1.0	-1.8	-1.8	-1.7
19		3R6X	-1.9	-1.9	-1.9	-2.2	-1.8	-2.0	-1.1	-1.7	-1.8	-1.6
20		4WG4	-0.6	-0.5	-0.7	-0.7	-0.6	-0.5	0.0	-0.6	-0.5	-0.5
21		1PXN	-0.7	-0.7	-0.9	-0.9	-0.8	-0.7	0.0	-0.7	-0.6	-0.5
22		3ROY	-2.4	-2.2	-2.3	-2.5	-2.3	-2.4	-1.7	-2.2	-2.3	-2.1
23		2C6O	-1.1	-0.9	-1.2	-1.2	-1.1	-1.0	-0.4	-1.1	-1.0	-1.0
24		3RAK	-1.7	-1.6	-1.7	-1.9	-1.7	-1.7	-0.9	-1.5	-1.6	-1.4
25		4EL9	-1.9	-2.2	-2.1	-2.5	-2.1	-2.0	-1.1	-1.7	-1.9	-1.6
26	Cation-π	1PXL	-6.5	-6.5	-6.6	-6.6	-6.6	-6.6	-6.3	-6.7	-6.6	-6.6
27		1FGI	-4.4	-4.8	-4.9	-5.0	-4.5	-4.7	-3.7	-4.6	-4.5	-4.4

(Continued)

Table S3 *(Continued)*

No.	Interaction	PDB ID	CCSD(T)/ CBS	DFT/Def2-QZVP								
				BLYP	TPSS	B97	ωB97X	B3LYP	M062X	PW6B95	B2PLYP	PWPB95
28		1M2Q	-2.0	-2.7	-2.7	-2.9	-2.2	-2.4	-1.0	-2.2	-2.1	-1.9
29		2VGP	-1.5	-1.6	-1.7	-1.8	-1.6	-1.6	-1.4	-1.6	-1.5	-1.6
30		3OWP	-9.2	-10.0	-9.9	-10.3	-9.9	-9.9	-8.7	-9.6	-9.4	-9.3
31		3SQQ	-2.1	-2.3	-2.4	-2.6	-2.2	-2.3	-1.3	-2.0	-2.0	-1.8
32		3RJC	-2.1	-2.4	-2.4	-2.6	-2.2	-2.3	-1.5	-2.1	-2.1	-1.9
33		3QTW	-8.4	-9.8	-9.4	-9.9	-8.4	-9.4	-8.0	-8.7	-8.7	-8.4
34	H-Bonding	3SW4	-6.0	-4.6	-4.5	-3.9	-4.0	-5.2	-3.2	-3.4	-4.8	-3.5
35		3ZLY	-4.9	-4.0	-3.9	-3.5	-3.5	-4.2	-2.7	-3.1	-4.1	-3.2
36		3RZB	-7.0	-6.2	-5.8	-5.6	-5.5	-6.3	-4.8	-5.0	-6.1	-4.9
37		3RPY	-7.0	-5.7	-5.3	-4.9	-5.2	-6.0	-4.6	-4.8	-5.9	-4.9
38		3R28	-4.8	-3.2	-2.9	-2.4	-2.8	-3.6	-2.6	-2.6	-3.5	-2.6
39		3QZI	-4.6	-3.6	-3.2	-2.8	-3.1	-3.9	-3.0	-3.1	-3.8	-3.0
40		3SXF	-3.8	-2.8	-2.6	-2.3	-2.4	-3.1	-2.0	-2.1	-3.0	-2.1
41		2R3P	-6.1	-4.2	-3.9	-3.5	-3.8	-4.6	-3.5	-3.6	-4.6	-3.7
42	Salt bridge	3MA3	-111.0	-110.2	-110.0	-109.9	-110.4	-110.6	-110.2	-110.1	-110.1	-109.7
43		4L9I	-100.3	-101.1	-100.9	-100.6	-100.3	-100.8	-99.4	-99.9	-100.2	-99.5
44		1NVQ	-100.0	-99.0	-99.1	-98.6	-99.8	-99.6	-99.2	-98.9	-99.2	-98.7
45		4JIK	-115.9	-114.9	-115.0	-114.8	-115.6	-115.6	-115.1	-114.5	-115.2	-114.2
46		1BX6	-68.2	-69.8	-68.9	-69.2	-68.2	-68.2	-67.8	-68.2	-68.0	-68.0
47		4WG4	-92.6	-93.5	-92.9	-92.9	-92.5	-93.0	-92.2	-92.4	-92.4	-92.0
48		4MTA	-106.6	-106.6	-106.5	-106.2	-106.7	-107.0	-105.4	-105.8	-106.3	-105.4
49		1E9H	-101.5	-100.9	-100.3	-100.3	-101.2	-101.5	-101.3	-100.9	-100.9	-100.6
